# Research progress on the application of dual energy CT in gastric diseases

**DOI:** 10.3389/fmed.2026.1845195

**Published:** 2026-06-03

**Authors:** Lin-na Wu, Xin-yu Dai, Li-juan Wei

**Affiliations:** Department of Gastroenterology and Digestive Endoscopy Center, The Second Hospital of Jilin University, Changchun, Jilin, China

**Keywords:** dual energy CT, efficacy evaluation, gastric cancer, TNM staging, X-ray computed tomography

## Abstract

**Background and Objective:**

Early detection and precise preoperative staging of benign and malignant gastric tumors are crucial for tailoring personalized treatment strategies and improving patient prognosis. Dual-energy CT (DECT), through material decomposition techniques, provides multiparametric quantitative information. This enables a transition in gastric imaging from a single morphological assessment to a comprehensive “morphological and functional” evaluation. This review aims to systematically elaborate on the latest advances in the clinical application of DECT for gastric tumor evaluation and other gastric diseases.

**Main content:**

This article reviews recent literature, focusing on the progress of DECT in the diagnosis and treatment of gastric tumors, as well as clinical studies based on quantitative DECT parameters. Key areas covered include the improvement of early detection rates, differentiation of benign and malignant lesions, precise preoperative staging, and therapeutic response assessment.

**Conclusion:**

Dual-energy CT provides rich, multidimensional quantitative metrics for the comprehensive evaluation of gastric tumors, significantly enhancing diagnostic performance and precision. With hardware upgrades and deep integration with artificial intelligence technologies such as deep learning, DECT is poised to play a more central role in the precision medicine and longitudinal management of gastric cancer.

## 1 Introduction

Gastric cancer (GC) is a gastrointestinal malignancy with leading incidence and mortality rates worldwide. In patients with early GC, where lesions are confined to the mucosa and submucosa, the 5-years survival rate can exceed 95%; thus, prognosis is highly dependent on early diagnosis and precise clinical staging (1). Because benign mucosal lesions such as gastritis, peptic ulcers, and gastric bleeding also occur in the gastric mucosa, accurately differentiating early GC from these conditions is crucial (2). Gastrointestinal subepithelial lesions (SELs) are protuberant lesions covered by normal mucosal epithelium in the gastrointestinal tract. SELs are detected in approximately 1 in 300 endoscopic examinations, with two-thirds located in the stomach (3). Although the overall incidence of neoplastic SELs is lower than that of GC, their biological behavior varies drastically–ranging from lipomas with no malignant potential to gastrointestinal stromal tumors (GISTs) with invasive potential (4). Therefore, timely characterization and differential diagnosis are imperative.

The clinical gold standard for differentiating GC and other gastric diseases is digestive endoscopy combined with histopathological evaluation, which yields high diagnostic accuracy (5). However, endoscopy is invasive, and some early GCs have low contrast with the surrounding normal mucosa, leading to potential missed diagnoses. Furthermore, endoscopy cannot accurately assess the depth of gastric wall invasion or distant metastases of advanced malignant lesions (6). Therefore, achieving early detection, precise characterization, risk stratification, and therapeutic monitoring of gastric diseases via non-invasive imaging modalities is of great clinical significance. This review aims to analyze the core physical principles and quantitative parameter systems of DECT in gastric diseases, summarize the latest clinical applications in GC, SELs, and non-neoplastic lesions, and prospectively discuss the technical challenges and future prospects of its integration with radiomics and AI.

## 2 Basic principles of dual energy CT

Over the past two decades, the introduction of DECT technology has further expanded the physical boundaries of CT imaging. Conventional single-energy CT uses only a single X-ray spectrum, and the measured attenuation represents an integrated value across the entire spectrum, failing to reflect the energy dependence of specific tissue attenuation. By acquiring image datasets of the same scanning region at two different X-ray energy spectra, DECT can analyze the variations in material attenuation as a function of energy. The degree of X-ray attenuation by a material depends on its elemental composition (the linear attenuation coefficient μ increases with atomic number and physical density) and the photon energy level. Because different materials exhibit relatively specific attenuation changes under high- and low-energy X-ray spectra, DECT effectively overcomes the limitations of conventional CT in tissue characterization (7, 8). Leveraging the differences in attenuation coefficients of various materials (e.g., iodine, calcium, water) at different energy levels, DECT allows for precise material decomposition (9, 10).

For instance, subtracting the water component from contrast-enhanced CT images generates an iodine map. Iodine maps significantly improve the contrast between lesions and normal surrounding parenchyma, amplifying subtle attenuation differences and thereby facilitating the reliable detection and characterization of small lesions (11). Similarly, subtracting the iodine component generates a virtual non-contrast (VNC) image, which can replace a true unenhanced (TUE) scan in certain clinical scenarios (12). Although the noise level in VNC images may be slightly higher, leading to minor deviations in CT values compared with TUE scans, this difference is typically not clinically significant (13). Beyond maintaining diagnostic image quality, VNC imaging provides a substantial dosimetric benefit. Studies, such as the analysis by Sauter et al., have demonstrated that VNC images derived from contrast-enhanced phases can accurately replace TUE scans (14). By omitting the TUE phase, DECT can quantify a potential radiation dose reduction of up to 30%–50% in standard abdominal protocols (11). It is worth noting that in the specific context of gastric tumor evaluation, the portal venous phase often requires an extended scanning field from the diaphragmatic domes to the anal verge. Even under these extended anatomical requirements, substituting a triple-phase protocol with a dual-phase DECT approach (arterial and portal venous phases with VNC reconstruction) yields an average effective dose reduction of approximately 21.4% (15). Consequently, integrating VNC into routine clinical workflows significantly limits radiation exposure, remaining consistent with the ALARA (as low as reasonably achievable) principle. Furthermore, water-iodine separation technology provides a reliable means to quantify iodine concentration within tissues (16, 17).

Virtual monoenergetic images (VMIs) are reconstructed by DECT within the range of 40–190 keV, simulating the imaging effect of a monochromatic X-ray beam at a specific kiloelectron volt level. Low-energy VMIs (40–60 keV) significantly enhance tissue contrast, facilitating the detection of minute lesions, whereas high-energy VMIs effectively reduce metal artifacts. Regarding quantitative parameters, iodine concentration (IC) and normalized iodine concentration (NIC) have demonstrated good clinical utility in differentiating benign and malignant gastric lesions, detecting tumors, and risk stratification (18–20).

Normalized iodine concentration, calculated by determining the ratio of the lesion’s IC to the IC of the aorta or a vein at the same level, effectively eliminates the confounding effects of inter-individual hemodynamic variations and contrast injection protocols, yielding higher comparability. The effective atomic number (Zeff) reflects the atomic composition of tissues and can also be used for tumor characterization and benign-malignant differentiation (21). Additionally, the spectral attenuation curve reflects the characteristics of X-ray attenuation coefficients varying with energy. The slope of the spectral attenuation curve (λ_*HU*_) has been widely used in clinical practice. Previous studies have shown that λ_*HU*_ in the venous phase is positively correlated with the risk stratification of GISTs, which not only aids in benign-malignant differentiation but also serves as a potential non-invasive imaging biomarker for preoperatively estimating the mitotic count (22).

## 3 Materials and methods

### 3.1 Literature search strategy

A systematic literature search was conducted across the PubMed and Web of Science databases to identify relevant articles published over the past two decades (from January 2006 to March 2026). The search was restricted to peer-reviewed original research articles applying the Population, Intervention, Comparison, and Outcome (PICO) framework (23): (P) Population: patients with gastric diseases; (I) Intervention: DECT examination with image quality (IQ) assessment; (C) Comparison: other validated evaluation methods for specific conditions, such as conventional CT, surgical outcomes, or histopathological examination; and (O) Outcome: correlation of quantitative DECT parameters with validated evaluation metrics.

The PubMed search strategy comprised two main domains, utilizing Medical Subject Headings (MeSH) and text words (in titles and/or abstracts) combined with the Boolean operators “OR” and “AND.”

The first domain focused on DECT, incorporating the MeSH term “Radiography, Dual-Energy Scanned Projection,” and the MeSH term “Tomography, X-Ray Computed/methods” combined with the text word “dual-energy.” Additional text words included “DECT,” “dual-energy CT,” “spectral CT,” and “dual energy computed tomography.”

The second domain focused on gastrointestinal diseases, including the MeSH terms “Stomach Diseases” and “Stomach Neoplasms.”

### 3.2 Study selection

**FIGURE 1:**
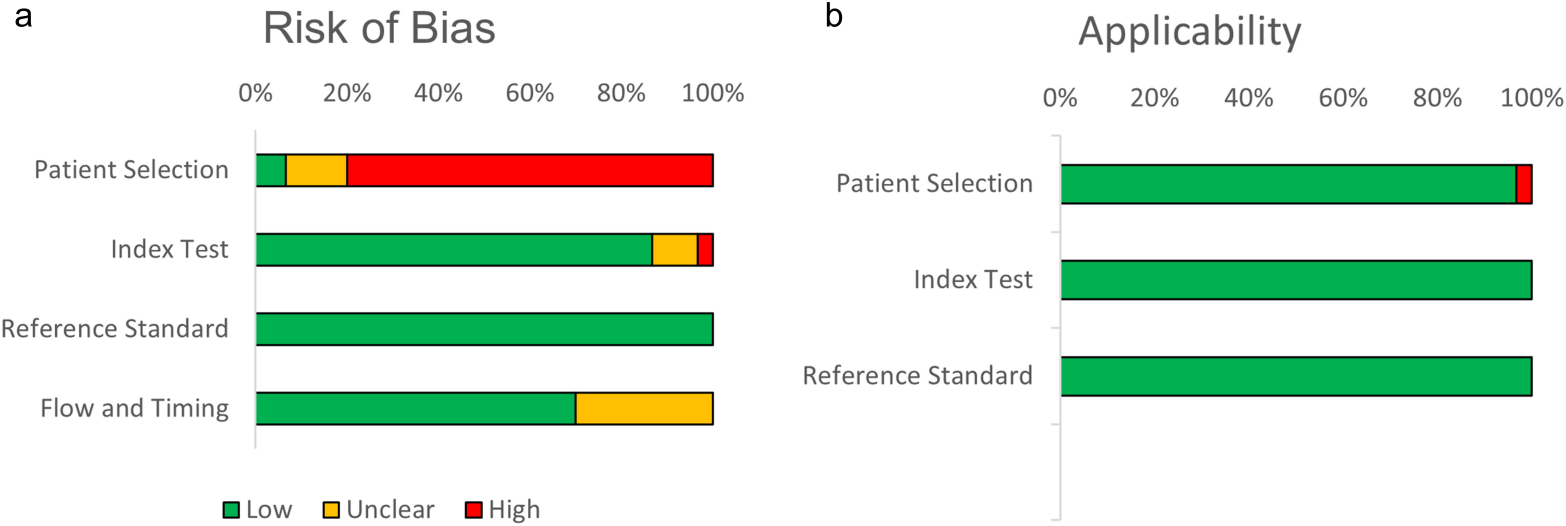
Bar chart labeled “Risk of Bias” **(a)** displays four assessment areas: Patient Selection, Index Test, Reference Standard, and Flow and Timing. Patient Selection shows majority high risk, minor unclear and low risk; Index Test shows mostly low risk with minor high and unclear; Reference Standard is entirely low risk; Flow and Timing is mostly low with some unclear risk. Bar chart labeled “Applicability” **(b)** shows all three categories—Patient Selection, Index Test, Reference Standard—are nearly entirely low risk, with Patient Selection having a minimal high risk portion. Color legend: green for low, yellow for unclear, red for high.

Two investigators independently performed the literature screening. Any discrepancies were resolved by a third investigator, guided by the following eligibility criteria. The inclusion criteria for this systematic review were limited to studies investigating the application of DECT in the stomach. Exclusion criteria were as follows: animal-only studies, non-English literature, review articles, case reports, and letters to the editor. The Quality Assessment of Diagnostic Accuracy Studies 2 (QUADAS-2) tool was used to assess the potential risk of bias (24) (Table 1). The research evidence was analyzed and organized through narrative and descriptive synthesis, mainly including the main research content, quantitative parameters used, and research results of the included literature. A total of 30 studies, published between 2013 and 2026, were ultimately included in this systematic review. Based on the QUADAS-2 assessment, the overall methodological quality of these studies was generally satisfactory. The majority of the studies demonstrated a low risk of bias across most domains. A comprehensive summary of the risk of bias and applicability concerns for all included studies is visualized in Figure 1.

**TABLE 1:** Risk of bias and applicability using the QUADAS-2 tool.

Study	Risk of bias				Applicability		
	Patient selection	Index test	Reference standard	Flow and timing	Patient selection	Index test	Reference standard
Chen et al. (43)	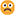	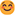	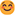	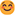	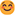	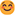	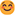
Liu et al. (44)	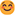	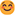	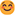	?	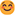	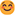	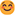
Lin et al. (48)	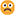	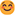	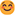	?	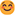	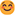	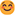
Li et al. (49)	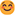	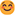	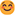	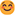	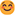	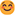	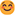
You et al. (33)	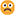	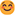	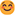	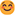	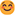	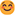	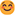
Lin et al. (47)	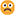	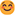	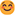	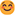	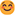	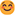	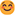
Li et al. (26)	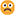	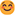	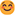	?	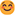	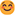	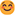
Chen et al. (76)	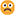	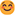	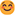	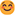	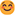	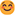	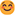
Li et al. (77)	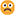	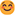	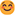	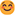	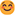	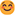	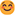
Yang et al. (31)	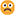	?	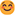	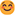	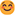	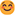	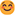
Liu et al. (32)	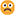	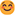	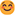	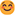	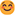	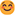	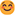
Feng et al. (78)	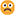	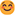	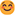	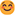	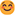	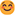	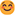
You et al. (79)	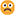	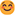	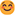	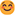	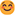	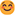	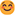
Li et al. (38)	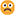	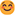	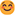	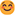	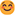	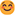	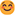
Yang et al. (80)	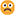	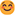	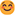	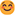	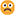	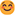	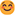
Liu et al. (50)	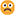	?	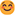	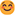	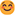	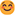	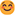
Chen et al. (81)	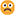	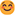	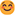	?	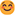	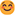	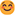
Chen et al. (82)	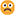	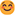	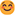	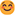	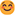	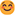	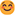
Zhao et al. (46)	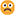	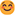	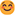	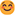	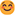	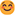	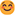
Li et al. (35)	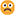	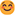	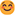	?	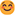	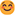	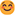
Küpeli et al. (30)	?	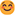	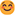	?	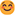	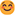	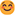
Gao et al. (83)	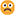	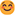	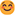	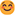	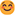	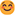	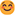
Yang et al. (84)	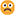	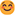	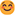	?	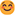	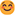	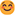
Liu et al. (42)	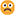	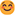	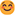	?	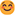	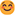	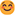
Meyer et al. (85)	?	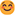	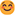	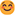	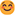	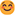	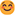
Tsurumaru et al. (57)	?	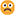	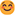	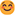	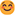	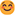	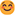
Wang et al. (60)	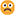	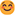	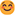	?	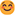	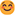	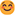
Meyer et al. (67)	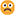	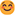	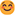	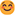	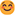	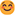	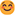
Meyer et al. (86)	?	?	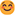	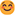	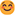	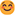	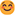
Baş and Zarbaliyev (71)	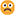	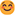	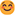	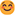	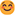	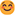	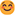

## 4 Results

**FIGURE 2:**
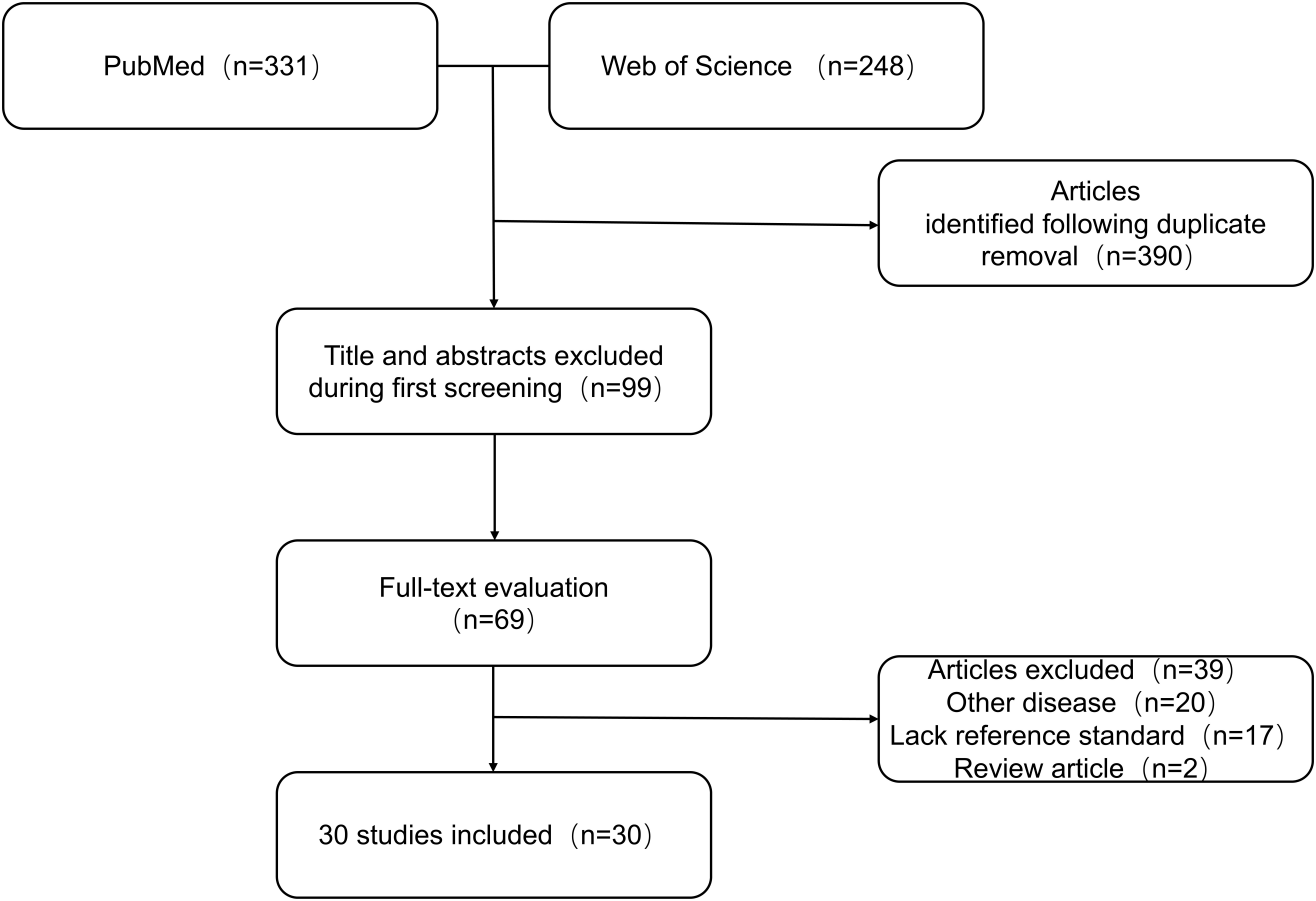
Flowchart showing the selection process for a systematic review: from 331 PubMed and 248 Web of Science records, 390 articles remained after duplicate removal. After screening, 99 were excluded, 69 underwent full-text evaluation, and 39 more were excluded. Thirty studies were included.

The initial database search yielded 579 studies. After the removal of duplicate records, 99 potentially relevant studies underwent screening based on title and abstract. Subsequently, 69 studies were selected for full-text evaluation. Following a comprehensive review applying the predefined inclusion and exclusion criteria, 30 studies were ultimately included in this review (Figure 2). Among these, 23 studies focused on gastric cancer (involving 4,523 patients), and 7 studies investigated benign gastric tumors and other gastric diseases (involving 370 patients).

### 4.1 Application of gastric cancer

Pathologically, early gastric cancer (EGC) is defined as a lesion where the depth of invasion is confined to the mucosa or submucosa, regardless of the presence of lymph node metastasis. Because early lesions often lack overt morphologic alterations, low-keV VMIs reconstructed from DECT data can exploit the high photoelectric effect of iodine to substantially amplify the attenuation of trace amounts of iodine contrast material accumulated within the gastric mucosa due to tumor angiogenesis. Consequently, even highly superficial EGC lesions can exhibit steep attenuation gradients compared with the surrounding normal submucosa or muscularis propria, thereby improving the image contrast-to-noise ratio (CNR) When combined with iodine mapping, areas of abnormal hypervascularity within the gastric wall can be readily identified, which significantly improves the detection rate of EGC (25).

Benign inflammatory mucosal lesions typically result from vasodilation, congestion, and transiently increased capillary permeability. Malignant mucosal lesions are characterized by the infiltration of neoplastic parenchymal cells and a proliferation of structurally abnormal, disorganized neovessels. A recent study evaluating the performance of quantitative DECT analysis in gastric mucosal lesions demonstrated significant differences in both IC and NIC between malignant and benign etiologies. Thus, quantitative DECT analysis serves as a valuable noninvasive adjunct for distinguishing malignant from benign gastric mucosal lesions (2) (Table 2).

**TABLE 2:** Application of DECT in gastric cancer.

References	Date	Population	Positive parameters	Results
Chen et al. (43)	2026	367	Quantitative parameters of VMI	The individualized model based on DECT virtual monoenergetic images effectively predicts Ki-67 expression status (AUC = 0.668.)
Liu et al. (44)	2026	459	Carbohydrate antigen 125 (CA125), nICVP, and λ_*HU*_(VP); cT, tumor thickness, location, and ICVP–ICAP (ΔICVP-AP)	Findings dual-energy CT-based nomograms demonstrated favorable performance in evaluating Ki-67 and PD-L1 expression status and were associated with response to immuno-chemotherapy in AGC (*p* < 0.05).
Lin et al. (48)	2025	115	ICDP-pre, ΔICAP, and tumor thickness-post	Findings sequential DECT-based ICDP-pre, ΔICAP, and tumor thickness-post were predictive of TRG status. Their combination demonstrated enhanced performance (AUC = 0.774, 95% CI: 0.686–0.846) and was associated with patient DFS. (95% CI: 1.004–4.991, *p* = 0.026).
Li et al. (49)	2025	317	Radiomics features extracted from polychromatic images, monochromatic (40 keV, 100 keV)	DECT radiomics demonstrates favorable performance in predicting NAC response (AUC = 0.806) and stratifying survival outcomes in LAGC. High-risk patients defined by DECT model had significantly worse overall survival (HR = 1.996, *p* = 0.012) and disease-free survival (HR = 1.873, *p* = 0.037) than low-risk counterparts.
You et al. (33)	2025	263	Tumor clinical T stage (cT), tumor thickness, venous-phase iodine concentration (ICVP), normalized arterial-phase iodine concentration (nICAP), and radscore (derived from logistic regression model).	His integrated model demonstrated favorable performance in the differentiation between T1/2 and T3/4a stage tumors in GC, with AUCs of 0.892 (95% CI: 0.829–0.956), 0.846 (95% CI: 0.734–0.958), 0.894 (95% CI: 0.818–0.970) and 0.821 (95% CI: 0.689–0.952) observed for the training, validation, external test 1, and external test 2 cohorts, respectively.
Lin et al. (47)	2025	54	%ΔIC-v, %ΔIC-d, %ΔNIC-v, %ΔNIC-d, %Δλ-v	Findings DECT parameters show higher preoperative predictive efficacy than RECIST 1.1 for TRG in gastric cancer (AUC = 0.877; 95% CI: 0.772–0.957 vs. AUC = 0.649; 95% CI: 0.496–0.803; *p* < 0.05)
Li et al. (26)	2025	51	keV VMIS	VMIs at 80–140 keV showed significantly fewer artifacts than composite images (*p* < 0.05).
Chen et al. (76)	2025	88	ECV, tumor area, and IDAV	DECT-derived ECV fraction is a valuable predictor of TRG in FAGC patients undergoing preoperative immuno-chemotherapy. (*p* < 0.05, OR = 0.911, 95% confidence interval, 0.798–0.994).
Li et al. (77)	2024	503	Radiomics features of polychromatic/monochromatic (40 keV, 100 keV)/iodine images	DECT radiomics can stratify patients preoperatively according to high-risk histopathologic phenotypes for gastric cancer (AUC > 0.80, *P* < 0.05)
Yang et al. (31)	2024	93	IC, NIC and CT value mean in VP and ROI area in LAP	DECT iodine-related parameters in VP are more predictive of the serosal invasion status of GC compared to LAP. (AUC = 0.805)
Liu et al. (32)	2024	342	IC, 70 keV, 100 keV monochromatic attenuations in the venous phase, and CT-reported T4a	The proposed quantitative model using multi-parameters in DECT accurately predicts serosal invasion for LAGC (AUC = 0.889) and showed a significant correlation with the DFS of patients (*p* < 0.05).
Feng et al. (78)	2024	219	NIC, IC and Zeff	The lower quantitative NIC, IC ratio, and Zeff on DECT was associated with peritoneal metastasis in advanced gastric cancer. (AUC = 0.884)
You et al. (79)	2024	179	Short-axis diameter, long-axis diameter, long-to-short-axis ratio, position, shape, density, edge, and the degree of enhancement, radiomics features of 120 kV linear fusion images	The models based on DECT LN radiomics features or combined traditional features have high diagnostic performance in determining the nature of each LN with a short-axis diameter of ≥6 mm in advanced GAC (AUC > 0.95)
Li et al. (38)	2023	240	Radiomics features of the IM and MIX images.	The R-COMB radiomics model based on DECT-IM and 120 kVp equivalent MIX images can effectively be used for preoperative noninvasive prediction of the Lauren classification of gastric cancer.
Yang et al. (80)	2022	231	Radiomics features of the 120-kV equivalent mixed images and iodine map (IM) images in the venous phase of DECT	The radiomics model (AUC = 0.9) and combined model constructed based on tumoral and peritumoral radiomics features derived from DECT (AUC = 0.93) showed high diagnostic efficacy for serosal invasion in gastric adenocarcinoma.
Liu et al. (50)	2021	69	Clinical stage, IC	He pre-treatment DECT-based clinical-radiomics nomogram showed good performance in predicting clinical response to systemic chemotherapy in AGC, which may contribute to clinical decision-making and improving patient survival. (AUC = 0.934)
Chen et al. (81)	2021	156	Iodine uptake images	DECT-derived radiomics serves as a promising non-invasive biomarker to predict survival for AGC patients after NAC, providing an opportunity for transforming proper treatment. (AUC = 0.78)
Chen et al. (82)	2021	239	Fourteen and three radiomics features with low redundancy and high importance were extracted from the IU and mixed images, respectively	DECT derived radiomics could serve as a non-invasive and easy-to-use biomarker to preoperatively predict PM for GC (AUC = 0.967, *p* = 0.528)
Zhao et al. (46)	2021	206	Tumor location, the iodine concentration of the tumor in the venous phase, and the normalized iodine concentration of the tumor in the venous phase	The DECT-based nomogram has great application potential in terms of detecting HER2 status in GC, and can serve as a novel substitute for invasive testing. (*P* < 0.05, AUC = 0.807, 95% CI, 0.718–0.897)
Li et al. (35)	2020	204	Radiomics features of images at arterial phase (AP) and venous phase (VP)	The DECT-based deep learning radiomics nomogram showed good performance in predicting LNM in gastric cancer (AUC = 0.84).
Küpeli et al. (30)	2019	41	Perigastric fat tissue	The IC in the perigastric fat tissue seems to be a reliable indicator for serosal invasion of gastric cancer. (*P* < 0.001)
Gao et al. (83)	2018	44	Total iodine uptake of portal phase (%ΔTIU-p)	The TIU-p can help predict pathological regression in advanced gastric cancer patients after neoadjuvant chemotherapy.
Yang et al. (84)	2018	43	ICPAT, SICPAT, ΔICPAT 和ΔSICPAT	These results showed that SICPAT is a reliable index for identifying post-NAC SI. (*p* < 0.05)

#### 4.1.1 Staging of gastric cancer

Prognosis and treatment decisions for gastric cancer rely heavily on precise TNM staging. Accurate T staging dictates the choice between neoadjuvant chemotherapy and upfront surgery, whereas N staging directly guides the extent of lymphadenectomy (e.g., D1 or D2 dissection). By providing both quantitative and qualitative assessments, DECT has emerged as a pivotal imaging modality for the accurate preoperative staging and prognostic evaluation of gastric cancer.

Current research paradigms are shifting from single-parameter analyses to integrated modeling. Notably, radiomics models that incorporate iodine maps, quantitative spectral parameters, and conventional clinical features have demonstrated exceptional diagnostic performance in the preoperative identification of T4-stage tumors.

#### 4.1.2 T staging

Accurate T staging of GC depends on the precise assessment of transmural tumor invasion depth, particularly in identifying serosal involvement to differentiate T3 from T4a disease. Titanium clips are frequently placed endoscopically during biopsy or pre-operative marking for precise lesion localization. While these clips are crucial for guiding complete tumor resection, their resulting metallic artifacts can severely degrade the subsequent CT evaluation of EGC. To mitigate these metallic artifacts, Li et al. evaluated VMIs derived from both split-filter DECT (sfDECT) and dual-layer spectral CT (DLSCT) systems. Their results demonstrated a comparable trend in artifact reduction across both platforms. Specifically, platform-optimized VMIs (70–80 keV for sfDECT and 90–100 keV for DLSCT) effectively mitigate artifacts and preserve vascular contrast, thereby significantly improving lesion visibility and T-staging accuracy in patients with EGC (26). Furthermore, serosal invasion has been established as an independent risk factor for disease recurrence following curative gastrectomy for GC (27). Morphologically, T3 tumors typically present with fine, reticular soft-tissue stranding in the perigastric fat, while T4a tumors are characterized by dense, nodular, or band-like enhancing soft tissue extending to the visceral peritoneum, often obliterating the subserosal fat plane (28). The precise radiological distinction between T3 and T4a is pivotal for surgical planning and patient selection for neoadjuvant therapy. While some T3 cases may proceed to upfront D2 gastrectomy, the confirmation of T4a disease strongly mandates the administration of neoadjuvant chemotherapy (NAC) to downstage the tumor, achieve an R0 resection, and prevent peritoneal micrometastasis (29). Küpeli et al. (30) demonstrated that the IC of perigastric adipose tissue measured during the arterial and portal venous phases can be utilized to evaluate serosal invasion. By amplifying the intrinsic attenuation differences between the neoplastic lesion and the surrounding perigastric fat, iodine maps and low-keV VMIs generated by DECT clearly delineate the margin of extraserosal invasion. Yang et al. (31) reported significant differences in both arterial and venous phase IC and NIC when distinguishing between T1–T3 and T4a GC. Given the heterogeneity among different WHO histologic subtypes, iodine-related DECT parameters acquired in the venous phase (VP) proved more efficacious in predicting GC serosal invasion than those obtained in the late arterial phase (LAP). Building on this concept, Liu et al. (32) developed a quantitative model for predicting GC serosal invasion incorporating venous phase IC, 70-keV and 100-keV monoenergetic attenuation values, and CT-reported T4a status. This DECT-based quantitative model exhibited a significant positive correlation with pathologic T stage and was predictive of postoperative disease-free survival (DFS). Finally, You et al. (33) integrated a radiomics model based on DECT iodine maps, quantitative spectral parameters, and conventional clinical features, which demonstrated robust diagnostic performance in the preoperative differentiation of T3 and T4a stage tumors. DECT demonstrated robust diagnostic performance in the preoperative differentiation of T3 and T4a stage tumors, thereby providing surgeons with reliable imaging evidence to formulate optimal, individualized treatment strategies. By demonstrating robust diagnostic performance in the preoperative differentiation of T3 and T4a tumors, DECT equips surgeons with definitive imaging evidence essential for tailoring optimal and individualized operative strategies.

#### 4.1.3 N staging

Accurate preoperative N staging remains a persistent challenge across various imaging modalities. A comparative study evaluating the diagnostic accuracy of DECT versus multiparametric MRI (mpMRI) for gastric cancer staging revealed that mpMRI yielded an 8.9%–12.9% higher overall accuracy for T staging compared with DECT. Furthermore, the overall accuracy of DECT for N staging was also shown to be inferior to that of mpMRI (34). However, recent advancements utilizing DECT-derived deep learning radiomics models have demonstrated robust diagnostic performance in predicting lymph node metastasis in patients with GC, highlighting a promising avenue for improving noninvasive N staging (35).

### 4.2 Lauren classification and differentiation prediction of gastric cancer

According to the Laurén classification system, GC is categorized into intestinal, diffuse, and mixed subtypes (36). Among these, the diffuse subtype exhibits a higher degree of malignancy and poor cellular adhesion. Consequently, it is highly prone to occult peritoneal dissemination and is associated with a high rate of postoperative recurrence (37). In a large retrospective cohort comprising 240 patients with GC, investigators successfully developed a combined radiomics (R-COMB) model to predict the Laurén classification. This model extracted radiomics features from venous-phase DECT iodine maps and 120-kVp linearly blended images. The model yielded an overall accuracy of 80.3% in the test set. Notably, this performance substantially outperformed the 64.7% concordance rate achieved by preoperative endoscopic biopsy during the same period (38).

### 4.3 Prediction of expression of gastric cancer biomarkers

The Ki-67 antigen is a nuclear protein expressed during all active phases of the cell cycle. High Ki-67 expression typically correlates with rapid tumor proliferation, hypervascularity, and aggressive biological behavior (39). Numerous studies have demonstrated that venous and delayed-phase IC and NIC, as well as the slopes of the λ_*HU*_ derived from DECT, are significantly and positively correlated with the Ki-67 labeling index determined via immunohistochemistry (40–42). Furthermore, Chen et al. (43) utilized a machine learning model based on DECT VMIs to effectively predict Ki-67 expression status, providing a noninvasive tool for prognostic risk stratification in patients with GC. Recently, a multicenter study developed an evaluation model to predict the expression of Ki-67 and the immune checkpoint programmed cell death ligand 1 (PD-L1) in advanced GC, integrating clinical features with quantitative DECT parameters. This model not only facilitates the noninvasive preoperative assessment of Ki-67 and PD-L1 status but also directly correlates with the treatment response in patients with advanced GC receiving combined chemoimmunotherapy, demonstrating robust predictive value (44).

Human epidermal growth factor receptor 2 (HER2) plays a pivotal role in gastric tumorigenesis, and patients with HER2-positive GC significantly benefit from HER2-targeted therapies (45). Currently, establishing HER2 status relies on immunohistochemical analysis of endoscopic biopsy or surgical resection specimens, a process that requires invasive tissue sampling and is time-intensive. To address this limitation, a noninvasive diagnostic model based on quantitative DECT parameter maps has been developed to identify HER2 expression status in GC, demonstrating promising clinical utility (46).

### 4.4 Monitoring of chemotherapy efficacy and prognostic evaluation for GC

Neoadjuvant chemotherapy combined with D2 gastrectomy has become the standard treatment paradigm for improving surgical resection rates and prolonging survival in patients with locally advanced gastric cancer (LAGC). Early and accurate evaluation of the response to NAC not only facilitates the timely discontinuation of ineffective chemotherapeutic regimens but also provides valuable guidance for tailoring personalized maintenance therapies. Currently, the reference standard for evaluating treatment response in solid tumors in clinical practice–the Response Evaluation Criteria in Solid Tumors, version 1.1 (RECIST 1.1)–relies primarily on temporal changes in one-dimensional morphologic measurements. However, Lin et al. (47) demonstrated that quantitative DECT parameters offer superior predictive value for pathologic tumor regression grade (TRG) in GC compared with RECIST 1.1. Their findings indicated that the rate of decline in venous-phase NIC was significantly steeper in treatment responders than in nonresponders, demonstrating that DECT-derived metrics can effectively assess GC treatment efficacy. Furthermore, in evaluating longitudinal pathologic response outcomes in patients with GC before and after neoadjuvant immunotherapy (NICT), subsequent studies (48) revealed that the rate of change in arterial-phase IC and the post-treatment delayed-phase IC were highly predictive of TRG and correlated significantly with DFS. By dynamically monitoring temporal changes in iodine uptake, DECT serves as a sensitive, noninvasive imaging biomarker for quantifying residual viable tumor burden following neoadjuvant therapy. The ability to accurately quantify viable tumor burden translates directly into enhanced systemic disease control. By identifying non-responders at an early stage, DECT allows oncologists to promptly pivot to second-line targeted therapies or expedite surgical intervention. This proactive approach prevents patients from enduring the unnecessary toxicity of ineffective regimens and strictly minimizes the window of opportunity for micrometastatic dissemination. Multiple studies have corroborated its substantial clinical value and research potential in this domain (49, 50), cementing DECT as a critical tool not merely for local assessment, but for the comprehensive management of systemic oncological disease.

### 4.5 Application of DECT in gastric submucosal tumors

Gastric submucosal tumors (SMTs) comprise a heterogeneous group of neoplasms arising beneath the gastric mucosa, predominantly of mesenchymal origin. While GISTs can arise anywhere along the gastrointestinal tract, the most frequent primary sites are the stomach (63%). Although the overall incidence shows no significant sex predilection (51), subtle differences may exist among specific subtypes. Gastric GISTs, particularly small lesions, share overlapping imaging features with other SMTs, including gastric leiomyomas, ectopic pancreas, schwannomas, and lipomas, rendering differential diagnosis highly challenging. Driven by advances in diagnostic and therapeutic modalities, the detection rate of these lesions has steadily increased in recent years.

As the primary screening modality for gastric diseases, conventional endoscopy allows for the direct visual assessment of tumor location, size, and overlying mucosal integrity (52). However, its diagnostic scope is strictly limited to the intraluminal protrusion; it cannot delineate the tumor’s layer of origin within the gastric wall, evaluate extraluminal extension, or depict internal tumor architecture. Consequently, macroscopic differentiation among GISTs, leiomyomas, and lipomas is virtually impossible. Furthermore, owing to their subepithelial location, routine superficial biopsies frequently yield false-negative results due to inadequate tissue sampling (53). Endoscopic ultrasonography (EUS) serves as a critical diagnostic adjunct, capable of delineating the mural layer of origin and evaluating sonographic features to assess tumor size, morphology, and depth (54). Despite its high diagnostic value, EUS remains an invasive procedure, and image quality can be compromised by intervening bowel gas. Crucially, for large tumors demonstrating significant exophytic growth, EUS struggles to capture the entire lesion and is inadequate for evaluating adjacent organ invasion or distant metastases (55). Although the European Society of Gastrointestinal Endoscopy (ESGE) recommends EUS as the premier modality for characterizing SMTs, EUS alone remains insufficient for definitively differentiating all SMT subtypes (52) (Table 3).

**TABLE 3:** Application of DECT in gastric submucosal tumors and other gastric diseases.

References	Date	Main research content	Population	Positive parameters	Results
Liu et al. (42)	2025	Predict the expression levels of GIST biomarkers	91	The venous phase enhanced CT iodine density maps and effective atomic number maps of DSCT	The nomogram model combining clinical features and radiomics (iodine density map radscore + effective atomic number map radscore) has the highest accuracy for preoperative prediction of Ki-67 expression in GISTs (AUC = 0.982, 95% CI, 0.9603–1)
Meyer et al. (85)	2024	Evaluation of chemotherapy efficacy of GIST	138	ViTB	DECT ViTB is a reliable response criteria and provides additional value for assessing TKI treatment in GIST patients. (*p* < 0.036)
Tsurumaru et al. (57)	2023	Identification of SMT: GIST and GL	26	NIC and λ_*HU*_	DECT parameters including NIC and λHU show promise as indicators for differentiating small-sized GISTs from leiomyomas. (*p* < 0.05)
Wang et al. (60)	2023	Identification of SMT: GIST and GS	45	Among the IMD histogram parameters of AP: Perc.50, Perc.90, Perc.99, variance, and skewness; among the IMD histogram parameters of VP: Perc.90, Perc.99, and the variance.	Histogram analysis based on IMD images obtained through DECT holds promise as a valuable tool for the preoperative distinction between GS and GST (≤5 cm) (all *P* < 0.05)
Meyer et al. (67)	2022	Evaluation of chemotherapy efficacy of GIST	40	ViTB	DECT vital iodine TB criteria showed performance comparable to that of EORTC PET criteria and outperformed RECIST 1.1 and mChoi criteria for response assessment of metastatic GIST treated with TKI therapy.
Meyer et al. (86)	2013	Efficacy evaluation of advanced GIST	17	ViTB	DECT allows a better prediction of therapeutic benefit in advanced GIST patients treated with tyrosine-kinase-inhibitors than established response criteria.
Baş and Zarbaliyev (71)	2021	Gastrointestinal emergencies	13	N/A	Conclusion DECT is significantly effective in planning surgical treatment and determining the foci of perforation in GIT perforations.

#### 4.5.1 Differential diagnosis

Gastrointestinal stromal tumors originate from the interstitial cells of Cajal (ICC) or their precursors within the gastrointestinal wall (56). They are characterized by KIT or PDGFRA gene mutations that drive abnormal capillary proliferation, resulting in highly hypervascular lesions. Through multiparametric DECT analysis, gastric GISTs can be reliably distinguished from other SMTs.

Conversely, gastric leiomyomas originate from the smooth muscle cells of the muscularis propria. They are characterized by densely packed cells and an abundant collagenous stroma; however, their vascular networks develop slowly, resulting in low microvessel density and a distinctly hypovascular appearance. Tsurumaru et al. (57) demonstrated that DECT parameters, notably NIC and λ_*HU*_, serve as robust quantitative markers for differentiating small GISTs from leiomyomas. In their cohort of small gastric SMTs (median diameter, 14.5 mm), DECT yielded high diagnostic performance, with sensitivities ranging from 77.8% to 88.9% and specificities from 76.5% to 100.0%. These findings surpass the diagnostic yield of conventional contrast-enhanced CT and provide crucial information prior to invasive tissue sampling. Furthermore, a recent study (58) reported that multiphase IC and λ_*HU*_ differed significantly (*P* < 0.01) between GISTs and other SMTs. Gastric schwannomas are extremely rare among gastric mesenchymal tumors, accounting for approximately 2% of cases, and originate from the Schwann cells of the myenteric (Auerbach) plexus. Clinically, they exhibit a benign biological course characterized by indolent growth, carrying an excellent prognosis following surgical resection with exceedingly rare recurrence or metastasis (59). Recent studies evaluating the texture and attenuation characteristics of DECT iodine maps have shown that arterial-phase iodine mapping can effectively differentiate GISTs from schwannomas. This discriminatory capability is closely tied to their underlying histopathologic divergence (60). Specifically, the dense proliferation of Schwann cells and robust collagen bundles in schwannomas produce a relatively homogeneous architecture that rarely undergoes necrosis or cystic degeneration, whereas GISTs are highly prone to such heterogeneous changes.

#### 4.5.2 GIST hazard stratification

Gastrointestinal stromal tumors possess malignant potential, making accurate risk stratification crucial for tailoring patient management. In clinical practice, the modified National Institutes of Health (NIH) risk stratification criteria represent the reference standard for histopathologic classification. Furthermore, the Ki-67 labeling index serves as a pivotal biomarker for evaluating the proliferative activity and aggressive biological behavior of GISTs.

Extensive literature demonstrates that venous-phase IC and NIC exhibit a significant positive correlation with the Ki-67 labeling index. This correlation arises because high-risk GISTs are characterized by rapid cellular proliferation and robust angiogenesis, which lead to increased intratumoral blood volume and, consequently, elevated iodine uptake. Thus, DECT-derived IC serves as a novel noninvasive imaging biomarker (61). Multiple studies have corroborated that these quantitative DECT parameters significantly improve the preoperative differentiation between high-risk and non-high-risk GISTs (62).

The integration of radiomics with DECT parameters represents a rapidly evolving frontier in clinical oncology. Several investigations have successfully extracted radiomic texture features from DECT iodine maps, demonstrating robust performance in evaluating abdominal malignancies (63). Although some evidence suggests that these high-order radiomic features may outperform basic quantitative DECT parameters, inherent variations in image acquisition, preprocessing, and feature extraction methodologies can limit the widespread clinical translatability of radiomics (64). Nevertheless, Liu et al. (42) recently developed and validated a DECT-based radiomics model utilizing venous-phase iodine maps and effective Zeff maps to predict Ki-67 expression levels in GISTs. This spectral radiomics model yielded an outstanding area under the receiver operating characteristic curve (AUC) of 0.982, substantially outperforming the AUC of 0.840 achieved by conventional contrast-enhanced CT radiomics models (65). These findings underscore the superior diagnostic performance of DECT-derived radiomics.

#### 4.5.3 Efficacy evaluation

For patients with unresectable, metastatic, or high-risk GISTs, molecular targeted therapy is the cornerstone of management. Imatinib, the first tyrosine kinase inhibitor (TKI) approved for GISTs, remains the first-line therapy and significantly prolongs overall survival. For patients who develop imatinib resistance, second- or third-line agents such as sunitinib or regorafenib are indicated.

Recent studies have underscored the value of DECT in this therapeutic landscape. An evaluation comparing true unenhanced (TUE) and VNC abdominal DECT images in patients with metastatic GISTs concluded that DECT represents a highly promising modality for assessing treatment response (66). Furthermore, a comparative study evaluating DECT, conventional CT, and FDG PET/CT in patients with metastatic GISTs undergoing TKI therapy demonstrated that DECT-derived viable iodine tumor burden (TB) metrics were equivalent to PET/CT criteria for evaluating therapeutic response. If the conventional RECIST 1.1 criteria are applied during the early phases of treatment (e.g., 1–3 months), they may fail to detect the marked reduction in tumor viability. Furthermore, they may inadvertently classify the response as “disease progression” due to a paradoxical increase in tumor size caused by intratumoral hemorrhage or myxoid degeneration. DECT overcomes these critical limitations by quantifying the “viable iodine tumor burden.” A pivotal study, combining an anthropomorphic phantom featuring spherical inserts of varying iodine concentrations with a retrospective clinical cohort, demonstrated that DECT provides exceptional sensitivity in capturing the precipitous decline in GIST angiogenesis following targeted therapy. The median progression-free survival (PFS) for patients classified as responders by DECT reached 623 days, compared with a mere 104 days for nonresponders, representing a highly significant difference (*P* < 0.001). This predictive performance significantly outperformed both the traditional RECIST 1.1 criteria and the modified Choi criteria (67). Ultimately, leveraging DECT material decomposition techniques to digitally subtract non-enhancing necrotic or cystic areas and precisely quantify the volume of physiologically perfused, viable tumor tissue has emerged as a cutting-edge functional imaging tool for monitoring targeted therapy in GISTs.

### 4.6 Exploration of DECT in other rare gastric tumors

Neuroendocrine neoplasms (NENs) originate from widely distributed endocrine cells derived from the endoderm, neuroectoderm, and neural crest. Gastric NENs account for approximately 3% of all gastric neoplasms, presenting with a highly diverse spectrum of clinical and biologic behaviors. Consequently, treatment strategies and patient prognosis are heavily dependent on tumor stage, primary anatomic site, and histologic grade (68). Emerging evidence (69) suggests that integrating various noninvasive qualitative and quantitative imaging modalities–including conventional CT, DECT, MRI, PET/CT, and somatostatin receptor (SSR) imaging–with novel radiotracers and texture analysis can significantly enhance the diagnostic workup. These advanced imaging techniques not only facilitate precise primary tumor staging and histologic grading but also aid in the prediction of biologic behavior.

### 4.7 Application of dual energy CT in non-tumor diseases of the stomach

Beyond its established role in evaluating gastric neoplasms, DECT provides significant clinical advantages in the emergency evaluation and longitudinal follow-up of acute non-neoplastic gastric conditions (e.g., gastrointestinal hemorrhage, perforation, and acute inflammation) and benign gastric ulcers.

Acute gastrointestinal (GI) bleeding is a common, potentially life-threatening emergency. Accurately localizing the culprit vessel and the precise site of active extravasation is a critical prerequisite for successful endoscopic hemostasis or transcatheter arterial embolization. By enabling the reconstruction of VNC images from contrast-enhanced datasets, DECT can obviate the need for true unenhanced acquisitions. This capability substantially reduces the cumulative radiation dose while preserving diagnostic accuracy. Furthermore, VNC images assist radiologists in reliably differentiating active bleeding from other hyperdense intraluminal material, such as ingested contents or hematomas (70). Complementarily, iodine mapping profoundly enhances the conspicuity of contrast material extravasation. This amplification improves the visibility of subtle bleeding sites, thereby increasing both the overall sensitivity and the localization accuracy for acute GI hemorrhage. Furthermore, in the context of acute gastric perforation, studies have reported a 100% concordance rate between the site of perforation identified on preoperative DECT and the actual transmural defect confirmed during subsequent surgical exploration (71).

## 5 Advantages and limitations of DECT

Dual-energy CT proves to be the superior imaging modality in clinical scenarios that demand simultaneous high-resolution anatomical mapping and functional tumor characterization. Specifically, DECT is highly advantageous in: (1) Early Detection and Accurate Staging of EGC: Utilizing low-keV (40 keV) monoenergetic algorithms to improve the visibility of subtle mucosal changes and decrease stage migration; (2) Early Treatment Response Evaluation: Detecting tumor devascularization and cystic degeneration through quantitative iodine maps in GIST patients undergoing TKI therapy, predicting progression-free survival more swiftly than conventional RECIST criteria; and (3) Spectral Differentiation of Submucosal Tumors: Accurately distinguishing specific gastric neoplasms (e.g., differentiating GISTs from leiomyomas) by analyzing spectral attenuation curves and effective atomic numbers, achieving a diagnostic confidence that SECT frequently cannot match. Despite the functional insights provided by DECT, its clinical implementation in gastrointestinal imaging entails specific technical and dosimetric limitations. A primary technical concern is the susceptibility to suboptimal image quality caused by photon starvation. This phenomenon is particularly pronounced at low X-ray energy spectrums when scanning patients with a large or protuberant abdomen. Consequently, to mitigate image noise, some institutions restrict DECT protocols to patients weighing less than 113 kg or to anatomical regions that can fit entirely within a 430 mm field of view (FOV) (72). From a dosimetric perspective, recent phantom-based simulations reveal that the radiation advantage of DECT is highly age- and size-dependent. While rapid kV-switching DECT can lower abdominal organ doses by 44%–45% in adult models compared to optimized single-energy CT (SECT), it paradoxically results in a 1.5- to 1.6-fold higher radiation burden in pediatric models. Thus, for pediatric or distinctly smaller patients, conventional SECT remains the more dose-efficient modality (73). Although gastric neoplasms are predominantly diseases of adulthood, this age-dependent dosimetric variability dictates that DECT cannot be universally applied across all demographic profiles. For pediatric or distinctly smaller patients requiring gastric or abdominal evaluation, conventional SECT remains the more dose-efficient modality, and DECT should be strictly reserved for complex cases where the functional spectral data is absolutely critical for an effective diagnosis. The accuracy of quantitative DECT parameters is highly susceptible to physiological and intraluminal gastric conditions. Adequate gastric distension is an absolute prerequisite for reliable spectral evaluation. Suboptimal distension leads to the pseudothickening of the gastric wall, which induces severe partial volume averaging effects. This phenomenon can artificially elevate local IC measurements, blurring the quantitative distinction between normal hyperemic mucosa and true neoplastic lesions, thereby increasing the risk of false positives or tumor overstaging. Similarly, the presence of retained food residue, mucous, or high-density intraluminal fluid introduces significant diagnostic challenges. Food particles can cause localized beam-hardening and streak artifacts that corrupt the underlying spectral data. Because dual-energy material decomposition algorithms rely on precise high- and low-energy attenuation profiles, these artifacts directly distort the calculations of IC, NIC and Zeff (74). Artifactual alterations in these metrics can either mimic the hypervascularity of a tumor or mask the subtle enhancement of an early mucosal lesion.

## 6 Future prospects

Despite the compelling diagnostic advantages of DECT in gastric oncology, its widespread integration into global clinical guidelines is currently hindered by the lack of standardized scanning protocols (75). The existing literature reveals significant heterogeneity in DECT acquisition parameters, including contrast media injection rates, scan delay times, and the selection of optimal energy levels for VMIs (49, 74). Furthermore, the intrinsic hardware differences among various DECT platforms–such as dual-source, rapid kVp-switching, and dual-layer detector systems–introduce inherent variations in spectral data processing and iodine quantification (18). To translate DECT from a research tool into routine clinical practice, it is imperative to establish internationally consensus-based protocols. Such standardization must include NIC calculation methods to ensure that quantitative spectral parameters are consistent, reproducible, and vendor-agnostic. Concurrently, future research must transition from single-center retrospective studies to robust, prospective, multicenter validations to ensure the broad generalizability of DECT-derived thresholds.

A major future trajectory for DECT involves integrating its high-quality spectral datasets with artificial intelligence (AI). Through advanced deep learning algorithms, this synergistic integration aims to achieve fully automated, accurate three-dimensional (3D) segmentation of the complex gastrointestinal lumen and thin gastric wall structures. However, the successful clinical deployment of these AI models is heavily bottlenecked by cross-vendor data heterogeneity. Variations in proprietary reconstruction algorithms, image noise textures, and spectral energy calibrations across different DECT platforms create significant “domain shifts.” These shifts severely degrade the generalizability of deep learning models, meaning an algorithm trained on data from one vendor may underperform on images from another. Moving forward, conducting multicenter, large-cohort studies and establishing high-quality, standardized imaging databases –equipped with robust cross-vendor image harmonization techniques–will be paramount. By deeply integrating quantitative DECT metrics with radiomics, and ensuring data standardization at the voxel level, researchers aim to ultimately realize the concept of a noninvasive “virtual biopsy” prior to surgical intervention. Ultimately, DECT is poised to become a core quantitative tool, facilitating global initiatives for gastric tumor screening, precise staging, and longitudinal treatment response monitoring.

## 7 Summary

In summary, DECT equips clinicians with robust and actionable quantitative parameters for the comprehensive management of gastric diseases. It overcomes the inherent diagnostic limitations of conventional single-energy CT, which has historically relied solely on morphologic dimensions and relative grayscale attenuation. By transcending purely anatomic delineation, DECT enables the precise quantification of microvascular function, hemodynamics, and intrinsic tissue composition. Consequently, it plays a pivotal role in precision medicine, facilitating personalized clinical decision-making and the tailoring of targeted interventions throughout the entire patient care continuum.
